# Meta-analysis of the efficiency and safety of neoadjuvant therapy with immune checkpoint inhibitors in resectable hepatocellular carcinoma

**DOI:** 10.3389/fmed.2024.1511511

**Published:** 2025-01-29

**Authors:** Adili Tuersun, Munire Mohetaer, Guanxin Hou, Gang Cheng

**Affiliations:** ^1^School of Life Sciences and Biopharmaceutics, Shenyang Pharmaceutical University, Shenyang, Liaoning, China; ^2^School of Business Administration, Shenyang Pharmaceutical University, Shenyang, China; ^3^Department of Pharmacy, General Hospital of Northern Theater Command, Shenyang, China; ^4^Department of Pharmacy, Shenyang Pharmaceutical University, Shenyang, China

**Keywords:** neoadjuvant immunotherapy, hepatocellular carcinoma, meta-analysis, safety, efficiency

## Abstract

**Purpose:**

Immunotherapy as a neoadjuvant treatment approach has achieved certain therapeutic effects in various types of cancer. However, in the specific cancer type of hepatocellular carcinoma (HCC), standardized protocols for neoadjuvant immunotherapy remain to be defined. This systematic review and meta-analysis focus on evaluating the efficacy and safety of neoadjuvant immunotherapy in the treatment of HCC, aiming to provide a robust basis for clinical decision-making.

**Methods:**

This study systematically searched databases such as PubMed, EMBASE, the Cochrane Library, and conference proceedings to identify clinical trials focusing on patients with HCC undergoing neoadjuvant immunotherapy. The Review Manager 5.4 software was applied to estimate the odds ratio (OR) of effect sizes and their corresponding 95% confidence intervals (CI).

**Results:**

Immune checkpoint inhibitors (ICIs) demonstrate significant efficacy in improving pathological outcomes and safety profiles in patients with resectable hepatocellular carcinoma (HCC). Specifically, ICIs significantly increase the pathological complete response (pCR) rate (OR = 0.23, 95% CI [0.14, 0.37], *p* < 0.00001) and major pathological response (MPR) rate (OR = 0.47, 95% CI [0.32, 0.70], *p* = 0.0002). They also markedly enhance the objective response rate (ORR) (OR = 0.42, 95% CI [0.28, 0.63], *p* < 0.0001). Furthermore, ICIs potentially improve the surgical resection rate (OR = 3.91, 95% CI [2.05, 7.45], *p* < 0.0001) and reduce the incidence of grade 3–4 treatment-related adverse events (TRAEs) (OR = 0.27, 95% CI [0.17, 0.44], *p* < 0.00001), indicating both therapeutic benefits and acceptable toxicity profiles.

**Conclusion:**

Neoadjuvant immunotherapy shows promise in the treatment of resectable HCC. Nonetheless, to further validate its efficacy, more large-scale, well-designed clinical trials are necessary to provide conclusive evidence.

**Systematic review registration:**

This comprehensive review adheres to the PRISMA (Preferred Reporting Items for Systematic Reviews and Meta-Analyses) standards and has been carried out as per a preregistered protocol (PROSPERO registration number: CRD42024560660).

## Highlights

- Neoadjuvant immunotherapy shows a 47% Major Pathological Response in resectable HCC.
- Complete Pathological Response achieved in 24% of patients post-neoadjuvant therapy.
- Objective Response Rate reaches 42% with neoadjuvant immunotherapy in HCC.
- Grade 3-4 Treatment-Related Adverse Event Rate is 27% in neoadjuvant immunotherapy.
- Systematic review emphasizes the need for large-scale trials to validate immunotherapy efficacy in HCC.

## 1 Introduction

Primary liver cancer (PLC) ranks sixth among malignant tumors and is the third leading cause of cancer-related deaths worldwide. Approximately 906,000 new cases emerge annually, with 830,000 fatalities (1). Currently, a variety of treatment modalities for hepatocellular carcinoma (HCC) are available, including surgical resection, liver transplantation, transarterial chemoembolization (TACE), ablation therapy, and pharmacological treatments. These therapeutic approaches collectively form an integrated treatment system aimed at providing personalized treatment plans. Despite surgical resection being an effective means for long-term survival, the majority of patients are diagnosed at an advanced stage, missing the optimal timing for surgery. Even with surgical intervention, the long-term survival rate for nearly 70% of patients remains poor, with a high risk of recurrence within 5 years. Therefore, improving the resection rate, reducing recurrence, establishing effective treatment plans and predictive indicators, and selecting patient subgroups likely to benefit have become key to treating HCC (2).

Immune checkpoint inhibitors (ICIs) are a class of targeted therapeutic drugs that act on specific receptors on the surface of cytotoxic T lymphocytes. These receptors include Cytotoxic T-Lymphocyte Associated Protein 4 (CTLA-4) and Programmed Cell Death Protein 1 (PD-1). ICIs enhance T-cell activity by blocking the interaction of these receptors with CD80/CD86 and PD-L1, thereby promoting an attack on tumor cells. Tumor cells often create an immunosuppressive microenvironment by upregulating the expression of immune checkpoint molecules to evade immune clearance (3). ICIs can block the interaction of these molecules with ligands on T cells, activating them to infiltrate tumor tissue and trigger cytotoxic T-cell responses, restoring antitumor immune reactions. In the early stages of HCC, the tumor microenvironment already exhibits immunosuppressive characteristics, such as an increase in regulatory T cells, a decline in the ratio of effector T cells to regulatory T cells, and a reduction in NK cells and dendritic cells. Studies have shown that an increase in CD4+ T cells is associated with prolonged recurrence-free time, memory CD8+ T cells are linked to a reduced risk of recurrence, and an increase in effector CD8+ T cells is correlated with an increased risk of recurrence. Additionally, an increase in PD-L1 expression levels is associated with shorter recurrence-free time (4, 5).

With enhanced screening for high-risk groups, an increasing number of early-stage HCC patients are being identified and given the opportunity for surgical resection. A phase II clinical trial by Kaseb et al. showed that preoperative treatment with nivolumab, either alone or in combination with ipilimumab, had good safety, with 25% of patients achieving pathological complete response (pCR) (6). A phase Ib clinical trial by Pinateo et al. confirmed the safety and tolerability of the combination therapy (7). Marron et al.’s study demonstrated that cemiplimab as neoadjuvant therapy can significantly promote tumor necrosis, supporting the application of neoadjuvant immunotherapy in HCC treatment (8).

Global liver cancer treatment guidelines have included immune checkpoint inhibitors, especially drugs targeting the PD-1/PD-L1 pathway, providing a new direction for neoadjuvant therapy in HCC. Neoadjuvant therapy shows potential to transform tumors into a “resectable cure” state, but there are still few clinical studies, and its efficacy and safety require further validation. A standardized protocol for neoadjuvant immunotherapy has not yet been established, and challenges remain in the standardization of treatment and patient selection. Therefore, in-depth research and clinical trials are crucial for determining the optimal application strategy and timing of neoadjuvant immunotherapy in the treatment of HCC.

## 2 Materials and methods

This comprehensive review adheres to the PRISMA (Preferred Reporting Items for Systematic Reviews and Meta-Analyses) standards (9) and has been carried out as per a preregistered protocol (PROSPERO registration number: CRD42024560660).

### 2.1 Search strategy

This study conducted a comprehensive search of the PubMed, EMBASE, and Cochrane Library electronic databases to gather relevant literature. To ensure the completeness of the materials, we also manually checked the reference lists of related literature and reviewed conference abstracts and reports, covering a time range from 2010 to May 2024. When conducting the literature search, we first identified key terms using Medical Subject Headings (MeSH) terminology and constructed a comprehensive search strategy using Boolean logical operators. The search keywords we used included “Hepatocellular Carcinoma (HCC),” “Neoadjuvant Therapy,” “Immune Checkpoint Inhibitors (ICIs),” as well as their synonyms and related derived terms. The specific search strategy, using PubMed as an example, is as follows:

**Table:** 

#1	“Randomized controlled trials as topic” [MeSH Terms] OR “random allocation” [MeSH Terms] OR “placebos” [MeSH Terms].
#2	“Carcinoma, Hepatocellular” [MeSH Terms] OR “Liver Cell Carcinoma, Adult” [MeSH Terms] OR “Liver Cancer, Adult” [MeSH Terms] OR “Adult Liver Cancer” [MeSH Terms] OR “Cancer, Adult Liver” [MeSH Terms] OR “Cancers, Adult Liver” [MeSH Terms] OR “Liver Cancers, Adult” [MeSH Terms] OR “Liver Cell Carcinoma” [MeSH Terms] OR “Carcinoma, Liver Cell” [MeSH Terms] OR “Carcinomas, Liver Cell” [MeSH Terms] OR “Cell Carcinoma, Liver” [MeSH Terms] OR “Cell Carcinomas, Liver” [MeSH Terms] OR “Hepatocellular Carcinoma” [MeSH Terms] OR “Hepatoma” [MeSH Terms].
#3	“Immune Checkpoint Inhibitors” [Title/Abstract] OR “Checkpoint Inhibitors, Immune” [Title/Abstract] OR “Immune Checkpoint Blockers” [Title/Abstract] OR “Checkpoint Blockers, Immune” [Title/Abstract] OR “PD L1 Inhibitors” [Title/Abstract] OR “Blockade, PD-1-PD-L1” [Title/Abstract] OR “PD 1 PD L1 Blockade” [Title/Abstract] OR “CTLA-4 Inhibitors” [Title/Abstract] OR “Cytotoxic T-Lymphocyte-Associated Protein 4 Inhibitors” [Title/Abstract] OR “Inhibitor, PD-1” [Title/Abstract].
#4	#1 AND #2 AND #3.

### 2.2 Study selection process

This study strictly adhered to the framework of Patients, Interventions, Comparisons, Outcomes, and Study design (PICOS) to establish the inclusion and exclusion criteria. The specific inclusion criteria are as follows:

1. Patients (P): The study subjects were patients diagnosed with resectable hepatocellular carcinoma (HCC), encompassing a diverse population without restrictions on gender or age. (2) Interventions (I): The study employed immune checkpoint inhibitors as neoadjuvant therapeutic agents.
2. Comparisons (C): This study did not specify a comparison group but focused on the effects of the single intervention.
3. Outcomes (O): The study must report at least one of the following primary outcome measures: Major Pathologic Response (MPR), Pathological Complete Response (pCR), the incidence of Grade 3–4 Treatment-Related Adverse Events (TRAEs) including liver function abnormalities, skin reactions, neurotoxicity, cardiotoxicity, infections, etc. (10), Objective Response Rate (ORR), and resection rate.
4. Study design (S): The study design must be an original research article, including single-arm or non-randomized controlled trials, to ensure the reliability and validity of the results.

The exclusion criteria are as follows:

1. The study subjects were patients with unresectable primary or metastatic HCC.
2. Participants had received immunotherapy or other systemic treatments prior to the study.
3. The literature was a duplicate publication of a research report.
4. The literature type was non-original research, such as reviews, meta-analyses, case reports, or case series.

### 2.3 Data extraction

This study involved two authors independently screening literature and extracting data to ensure the objectivity of the results. In cases of disagreement, a third author was involved to resolve the dispute. Subsequently, we conducted a comprehensive search of the selected articles and conducted an in-depth analysis of their full texts.

We meticulously recorded key information for each included study, including but not limited to: the first author and publication year; clinical trial registration number (NCT number); the intervention measures and treatment strategies used in the study; the type of article, including study design and methodology; the specific immune checkpoint inhibitor drugs used; the sample size of the study; and the primary outcome measures, including pCR, MPR, Grade 3–4 TRAEs, ORR, and resection rate.

Through this method, we ensured the completeness and traceability of the study data, providing a solid foundation for subsequent data analysis and interpretation of results.

### 2.4 Quality assessment of studies

To evaluate the quality of non-randomized studies, this research utilized the Methodological Index for Non-Randomized Studies (MINORS) as the assessment tool. This evaluation instrument includes a series of specific criteria such as the clarity of the study’s objectives, consistency in patient inclusion, anticipated data collection, appropriateness of endpoints reflecting the study’s objectives, objectivity in endpoint assessment, completeness of follow-up, dropout rate below 5%, and consideration of sample size estimation. Each criterion is scored on a scale from 0 to 2: 0 indicates not reported, 1 indicates reported but inadequate, and 2 indicates reported and fully detailed. This scoring mechanism ensures a meticulous assessment of the study quality, aiding in the identification of potential biases and limitations within the research. The specific scoring details and results are presented in Table 1.

**Table 1:** Methodological Index for Non-Randomized Studies (MINORS) assessment tool.

Author/year	A	B	C	D	E	F	G	H	I	G	K	L	Score
Shi, Y. H/2021 (12)	2	2	2	2	1	2	2	0	–	–	–	–	13
Su, Y/2021 (13)	2	2	2	2	2	2	2	0	–	–	–	–	14
Ho, W. J/2021 (14)	2	2	2	2	2	2	2	0	–	–	–	–	14
Marron/2022 (8)	2	2	2	2	1	2	2	0	–	–	–	–	13
Xia, Y/2022 (15)	2	2	2	2	2	2	2	0	–	–	–	–	14
Kaseb/2022 (6)	2	2	2	2	2	2	2	0	2	2	2	2	22
Chen, S/2022 (16)	2	2	2	2	1	2	2	0	–	–	–	–	13
Bai, X/2022 (17)	2	2	2	2	2	2	2	0	–	–	–	–	14
Song, T. Q/2023 (18)	2	2	2	2	1	2	2	0	–	–	–	–	13
D’Alessio/2023 (19)	2	2	2	2	2	2	2	0	–	–	–	–	14
Sun, H. C/2023 (20)	2	2	2	2	2	2	2	0	–	–	–	–	14
Wang, K (21)	2	2	2	2	2	2	2	0	2	2	2	2	22
Qin, S (22)	2	2	2	2	2	2	2	0	2	2	2	2	22
Kaseb, A (23)	2	2	2	2	2	2	2	0	2	2	2	2	22

A: The study’s aim is clearly stated. B: The coherence of patient inclusion is maintained. C: Data collection is conducted as expected. D: The endpoints appropriately reflect the study’s objectives. E: The evaluation of endpoints is objective. F: Sufficient follow-up time is ensured. G: The dropout rate is below 5%. H: Sample size estimation is performed. I: Additional criteria for evaluating studies with control groups. J: The selection of the control group is appropriate. K: The control group is contemporaneous. L: The baseline comparability between groups is ensured. M: Statistical analysis is conducted appropriately.

### 2.5 Data analysis

In this study, we utilized Review Manager 5.4 software for calculations and graphical representations. For single-arm studies lacking a control group, we employed an appropriate transformation method to handle binary outcome data, facilitating subsequent statistical analysis. The specific transformation process adheres to the following formula: *P* = ln(odds) = ln[X/(n-X)], where *n* represents the total sample size, *X* is the number of observed events, and *P* denotes the event occurrence rate. Furthermore, the standard error of the occurrence rate, SE(p), can be calculated using the formula: SE(p) = SE[ln(odds)] = [1/X + 1/n – X]^^1/2^ (11).

Based on the aforementioned transformation, we further estimated the Odds Ratio (OR) and its 95% Confidence Interval (CI) using the following formula: P_f_ = OR/1 + OR. Concurrently, the actual event occurrence rate P_f_ and its 95% CI were calculated using the formulas: LL = LLOR/1 + LLOR and UL = ULOR/1 + ULOR (11).

## 3 Results

### 3.1 Study selection process and results

**Figure 1:**
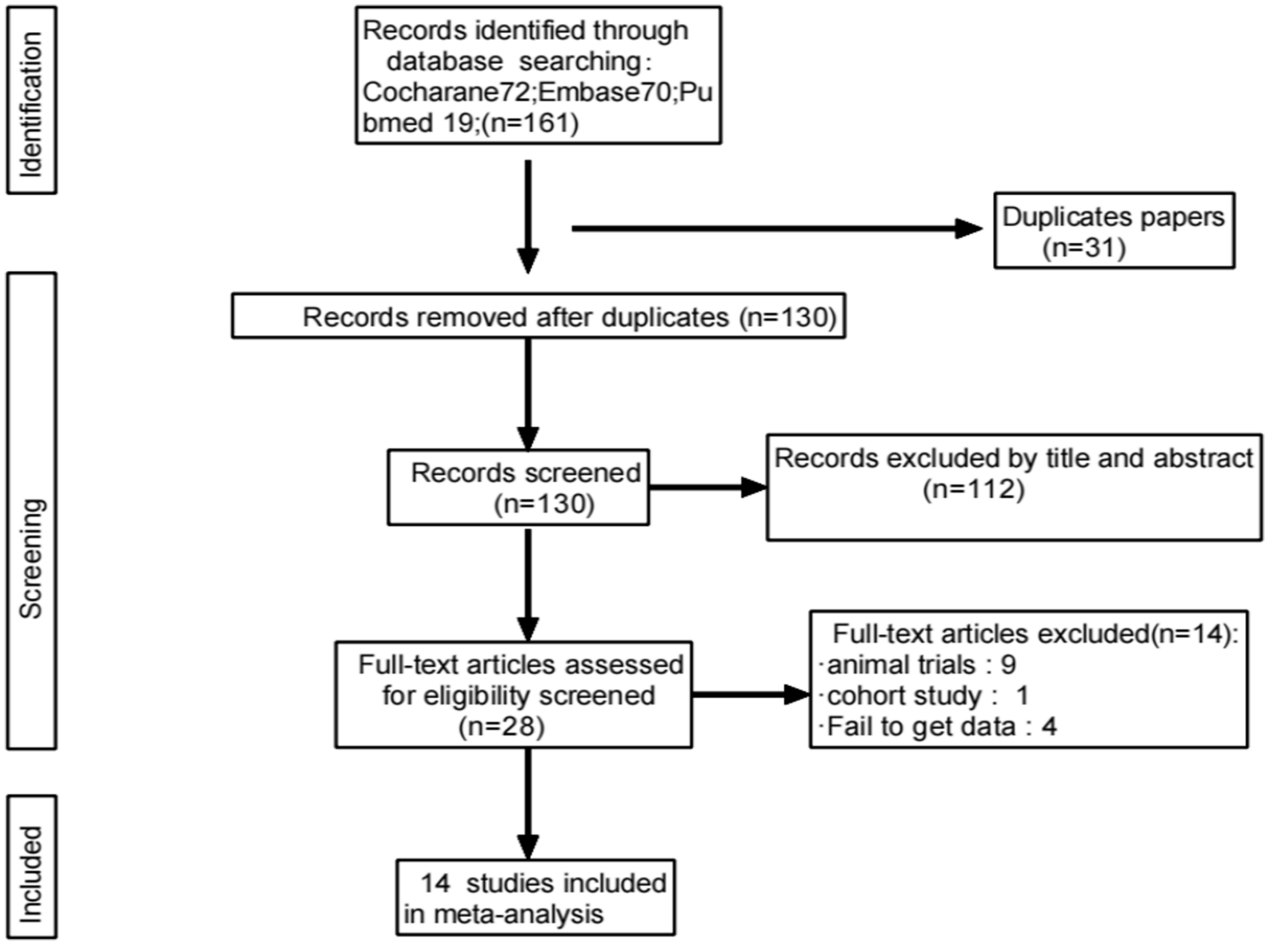
Flowchart of article selection.

A total of 161 relevant studies were initially identified. After removing duplicates, 130 studies were obtained. Following the review of titles and abstracts, 14 studies were ultimately included (6, 8, 12–23), as depicted in Figure 1.

### 3.2 Basic characteristics of included literature

A total of 14 articles were included in this study, covering a variety of neoadjuvant therapy drugs, including Tremelimumab, Nivolumab, Cemiplimab, and Camrelizumab. The incidence of pCR ranged from 5.9 to 38%, the incidence of MPR ranged from 17.6 to 56%, and the incidence of Grade 3–4 TRAEs ranged from 10 to 41.4%. The specific study characteristics and outcome data are detailed in Table 2.

**Table 2:** Characteristics of neoadjuvant immunotherapy studies in patients with hepatocellular carcinoma.

Author	Register number	Nation	Patient number	Neoadjuvant therapy	pCR (%)	MPR (%)	Grade 3–4 TRAEs (%)	ORR (%)	Resection rate (%)
Shi, Y. H (12)	NCT03867370	China	18	Tremelimumab/Tremelimumab + Lenvatinib	6.3	–	16.7	–	51.7
Su, Y (13)	NCT03510871	China	29	Nivolumab + Ipilimumab	–	33.3	41.4	–	80
Ho, W. J (14)	NCT03299946	USA	15	Nivolumab + Cabozantinib	8.3	33.3	13.3	–	95.2
Marron, T. U (8)	NCT03916627	USA	21	Cemiplimab	15	20	10	15	94.4
Xia, Y (15)	NCT04297202	China	20	Camrelizumab + Apatinib	5.9	17.6	16.7	16.7	74.1
Kaseb, A. O (6)	NCT03222076	USA	30	Nivolumab / Nivolumab + Ipilimumab	25	30	33	–	–
Chen, S (16)	NCT04615143	China	11	Tislelizumab	9.1	–	–	18.2	–
Bai, X (17)	NCT04930315	China	32	Camrelizumab + Apatinib	9.1	27.3	–	–	70.8
Song, T. Q (18)	NCT04834986	China	24	Tislelizumab + Lenvatinib	17.6	35.3	–	54.2	84
D’Alessio, A (19)	NCT03682276	UK	25	Nivolumab + Ipilimumab	38	56	–	29	56.7
Sun, H. C (20)	NCT04843943	China	30	Sintilimab + Bevacizumab	–	–	23.3	26.7	–
Wang, K (21)	ChiCTR2000037655	China	198	Sintilimab	–	–	12.4	–	–
Qin, S (22)	NCT04102098	China	668	Atezolizumab + Bevacizumab	–	–	27	–	87.5
Kaseb, A (23)	–	USA	14	Nivolumab + Ipilimumab	28.6	–	35.7	–	85.7

“–”: Not report.

### 3.3 Efficacy outcomes of immune checkpoint inhibitors

**Figure 2:**
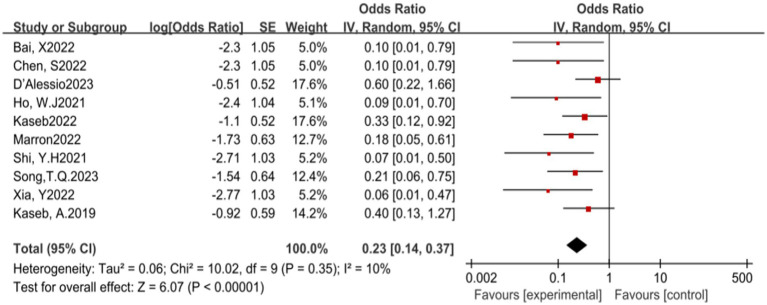
Forest plot of pCR outcomes with immune checkpoint inhibitors.

In this meta-analysis, a total of 10 studies reported the incidence of pCR. The results of the heterogeneity test indicated that there was no significant heterogeneity among the included studies (*p* = 0.35, *I^2^* = 10%). Further analysis revealed that ICIs have a statistically significant benefit in increasing the pCR rate, with an OR of 0.23, 95% CI (0.14, 0.37), and a *p*-value less than 0.00001, indicating a highly statistically significant effect (see Figure 2) for specifics.

**Figure 3:**
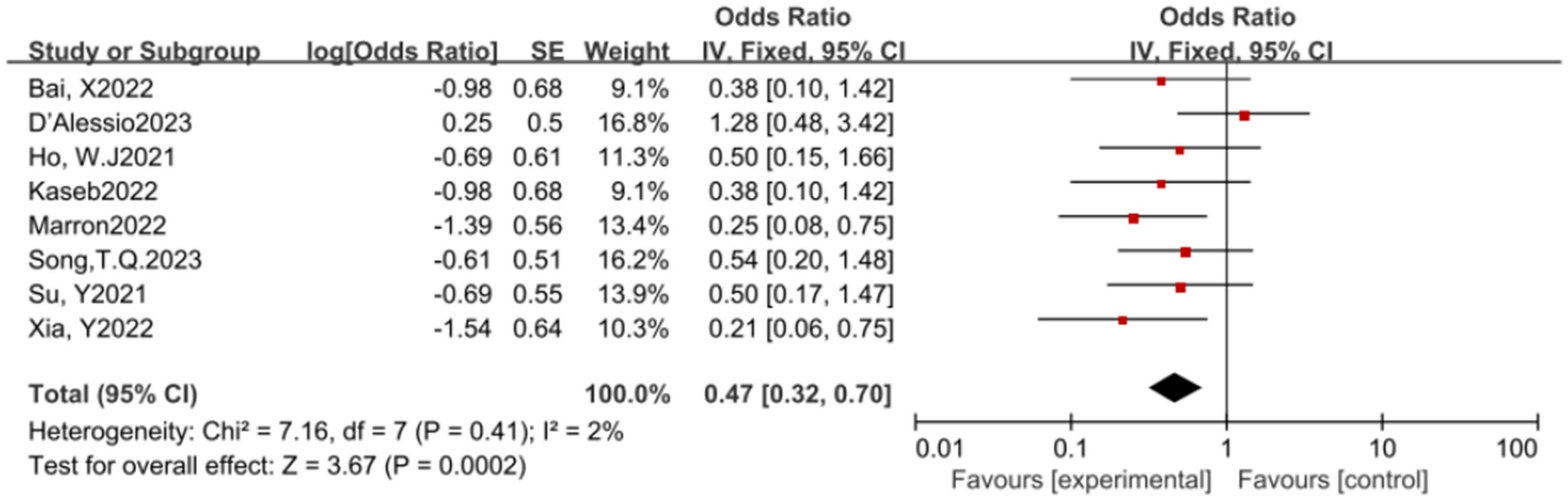
Forest plot of MPR outcomes with immune checkpoint inhibitors.

In this meta-analysis, a total of 8 studies reported the MPR rate. The results of the heterogeneity test indicated that there was no significant heterogeneity among these studies (*p* = 0.41, *I^2^* = 2%). A pooled analysis of these studies revealed that ICIs have a statistically significant benefit in increasing the MPR rate, with an OR of 0.47, 95% CI (0.32, 0.70), and a *p*-value of 0.0002, indicating a statistically significant effect of ICIs in promoting MPR (see Figure 3) for specifics.

**Figure 4:**
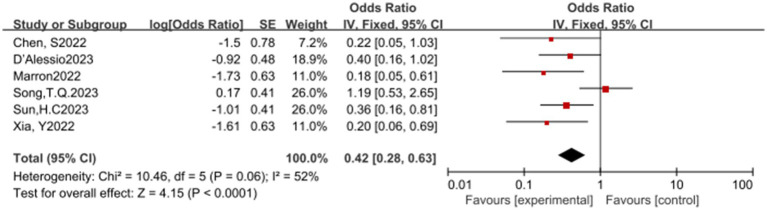
Forest plot of ORR outcomes with immune checkpoint inhibitors.

In this meta-analysis, a total of 6 studies reported the overall response rate (ORR). The results of the heterogeneity test showed significant heterogeneity (*p* = 0.06, *I^2^* = 52%), suggesting that caution should be exercised when interpreting the results. Despite the heterogeneity, the pooled analysis indicated that ICIs have a significant statistical benefit in increasing the ORR, with an OR of 0.42, 95% CI (0.28, 0.63), and a *p*-value less than 0.0001. This result suggests that ICIs have a potential therapeutic effect in promoting ORR (see Figure 4) for specifics.

### 3.4 Safety outcomes of immune checkpoint inhibitors

**Figure 5:**
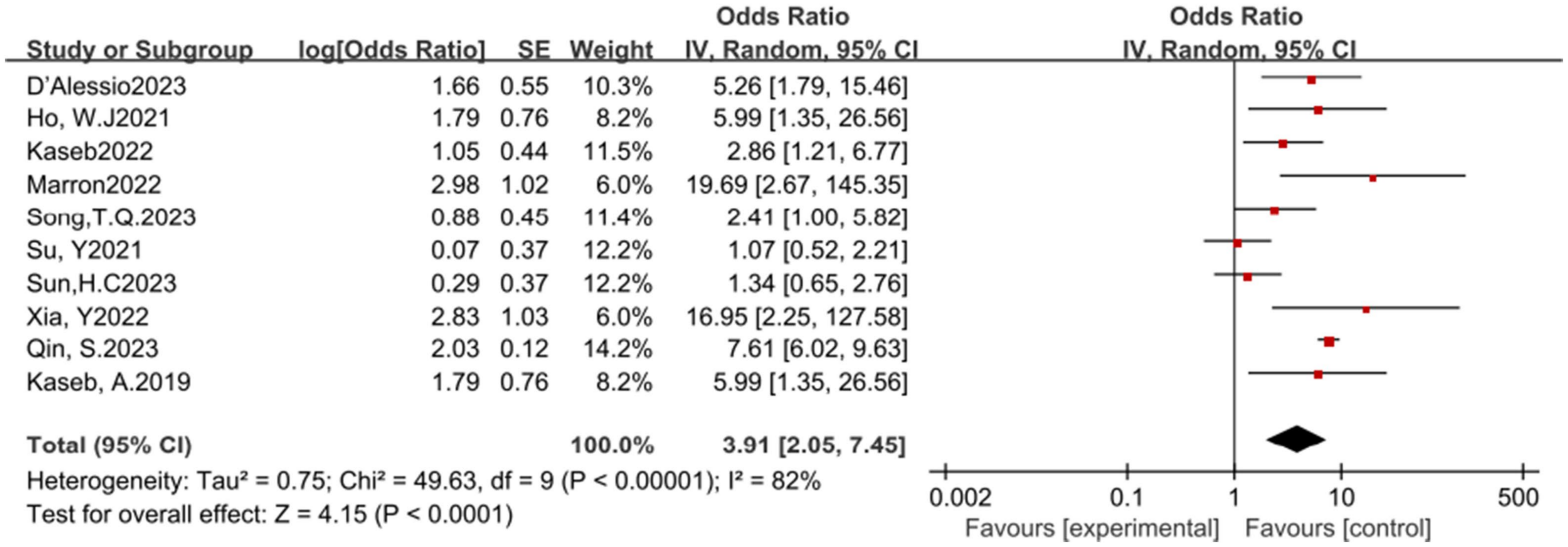
Forest plot of surgical resection rate outcomes with immune checkpoint inhibitors.

In this meta-analysis, a total of 10 studies reported the surgical resection rate. The results of the heterogeneity test showed significant heterogeneity (*p* < 0.00001, *I^2^* = 82%). Despite the significant heterogeneity, the pooled analysis results indicate that ICIs have a potential benefit in increasing the surgical resection rate, with an OR of 3.91, 95% CI (2.05, 7.45), and a *p*-value less than 0.0001 (see Figure 5) for specifics.

**Figure 6:**
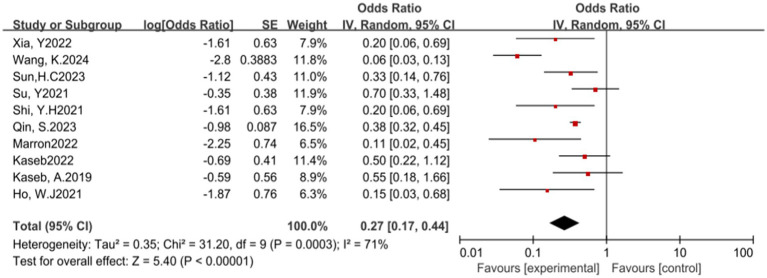
Forest plot of grade 3–4 treatment-related adverse events (TRAEs) outcomes with immune checkpoint inhibitors.

Additionally, the use of ICIs is closely associated with the incidence of treatment-related adverse events (TRAEs). A total of 10 studies reported the incidence of grade 3–4 TRAEs. The results of the heterogeneity test showed significant heterogeneity (*p* = 0.0003, *I^2^* = 71%). The pooled analysis results suggest that ICIs have a potential benefit in reducing the incidence of grade 3–4 TRAEs, with an OR of 0.27, 95% CI (0.17, 0.44), and a *p*-value less than 0.00001 (see Figure 6) for specifics.

### 3.5 Subgroup analysis

Subgroup analyses were conducted to assess the impact of various treatment regimens on clinical outcomes, including pCR, MPR, ORR, TRAEs, and surgical resection rates. This evaluation aimed to determine the differential effects of treatments on efficacy and safety profiles and their overall influence on patient outcomes.

**Figure 7:**
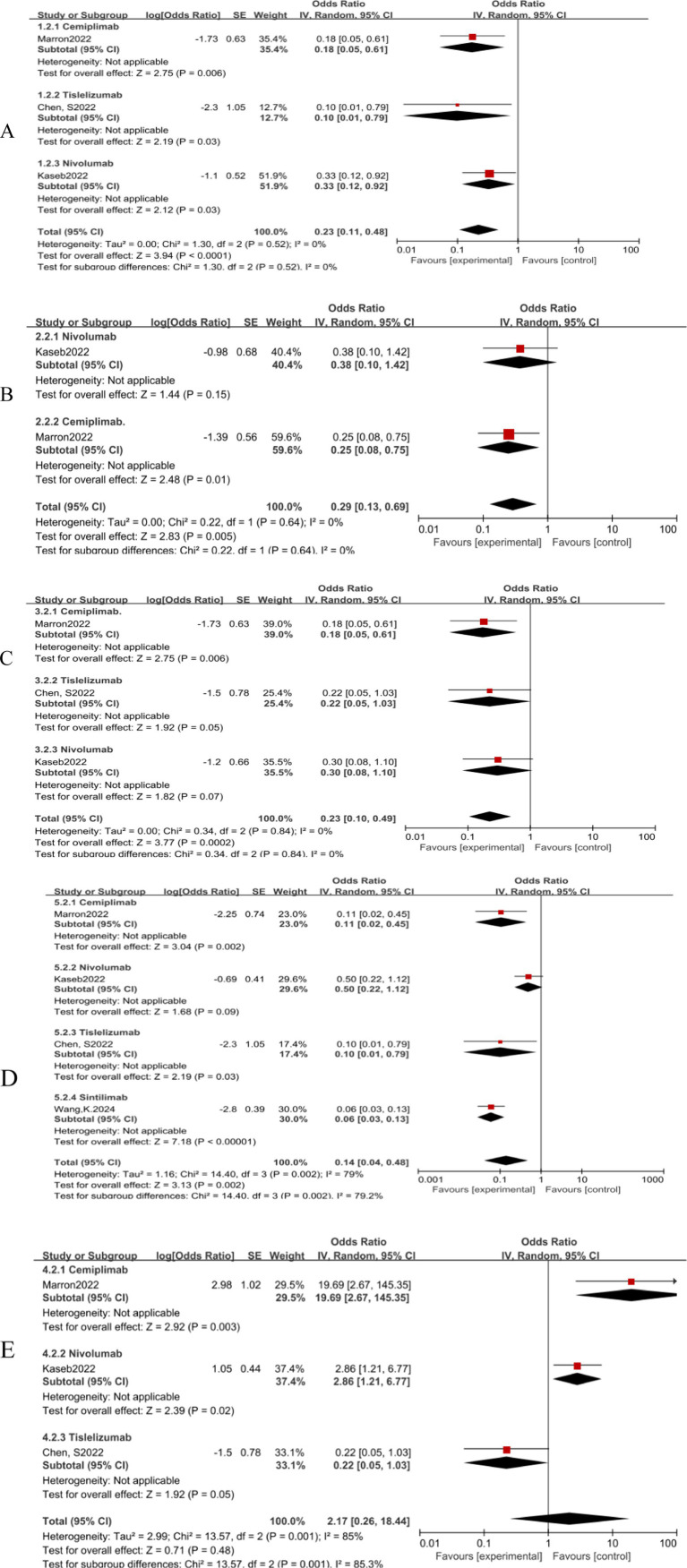
Subgroup analyses of immune checkpoint inhibitor drug types for **(A)** pCR, **(B)** MPR, **(C)** ORR, **(D)** Grade3–4 TRAEs and **(E)** Resection Rate. pCR, pathological complete response; MPR, major pathological response; ORR, Overall Response Rate; TRAEs, treatment-related adverse events.

The subgroup analysis revealed no significant efficacy differences between the three immune checkpoint inhibitors (ICIs) when used as monotherapies (Figures 7A–C). In terms of safety, nivolumab monotherapy (Figure 7D) exhibited a higher OR for adverse events compared to Cemiplimab, indicating a statistically significant difference. Conversely, Cemiplimab monotherapy showed a higher OR for the rate of excision (Figure 7E) than nivolumab, the difference was statistically significant.

**Figure 8:**
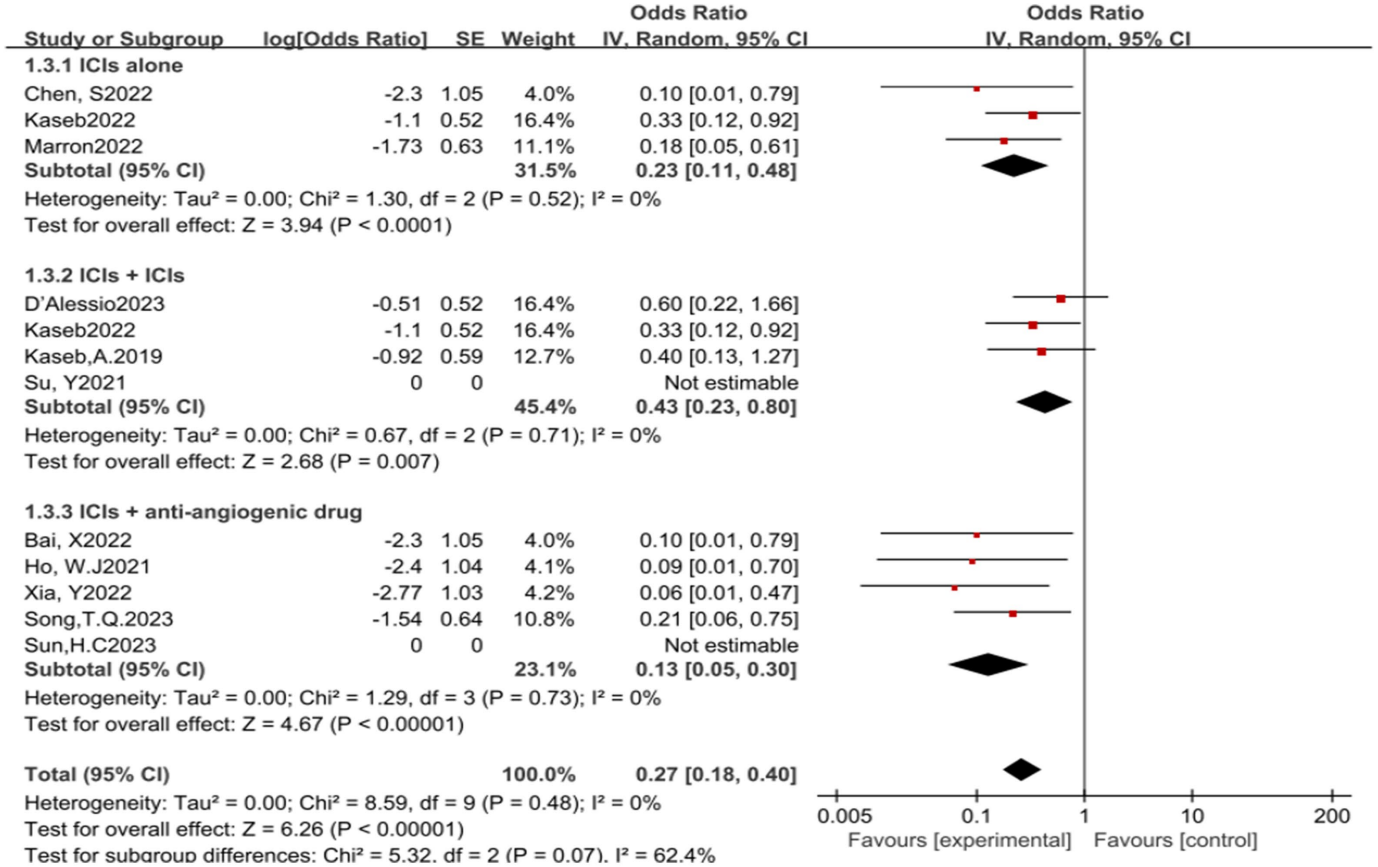
Subgroup analyses based on neoadjuvant immune checkpoint inhibitor combinations for pCR. pCR, pathological complete response.

**Figure 9:**
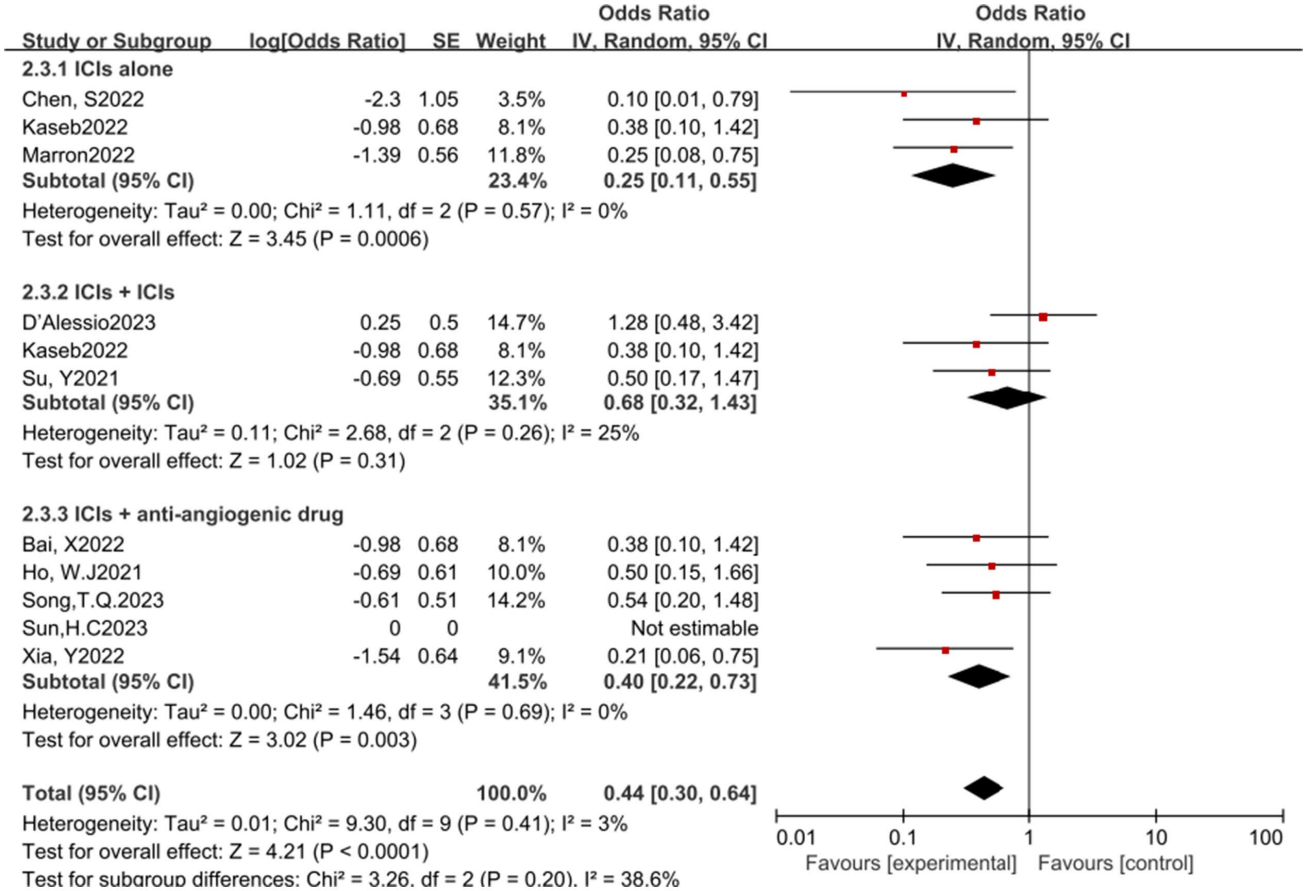
Subgroup analyses based on neoadjuvant immune checkpoint inhibitor combinations for MPR. MPR, major pathological response.

**Figure 10:**
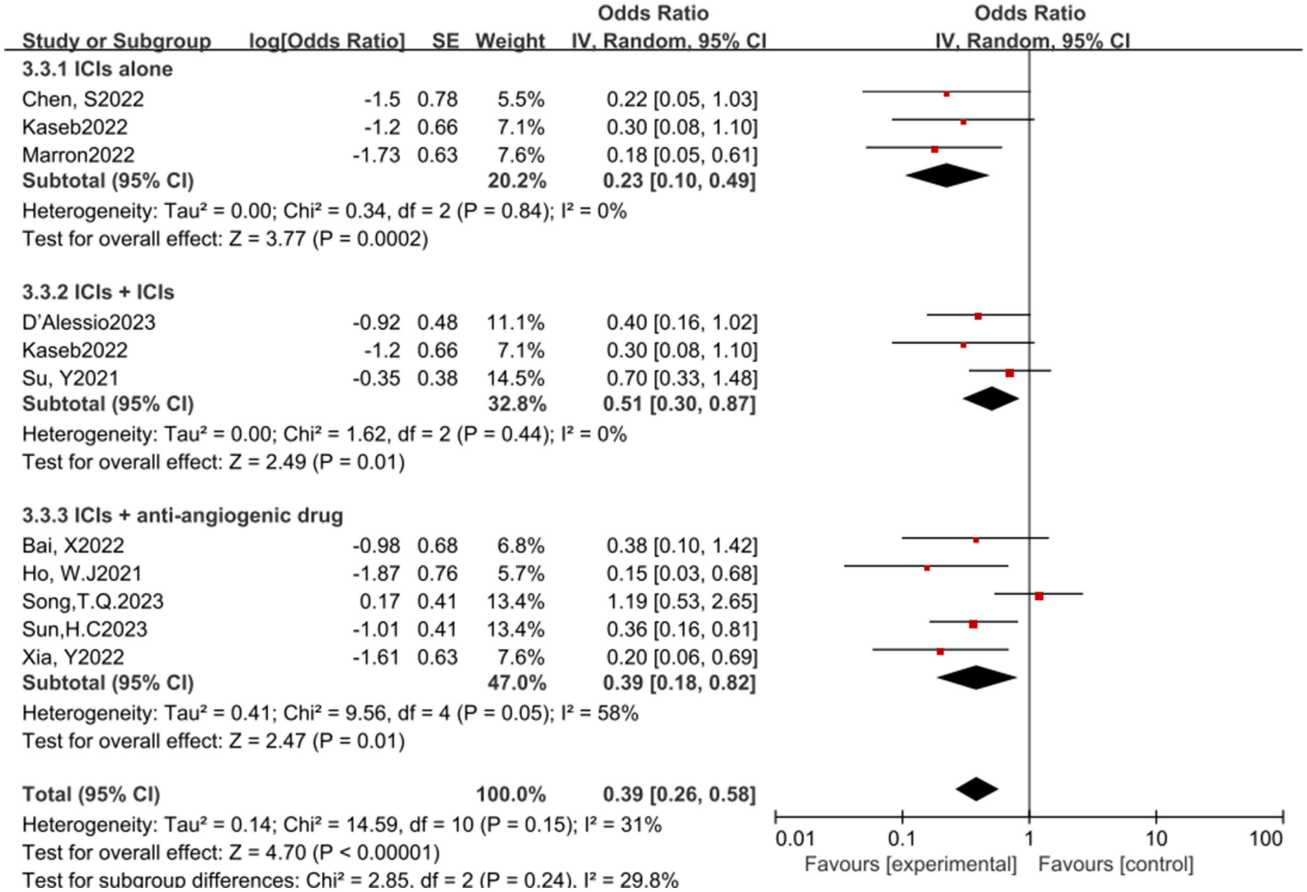
Subgroup analyses based on neoadjuvant immune checkpoint inhibitor combinations for ORR. ORR, Overall Response Rate.

**Figure 11:**
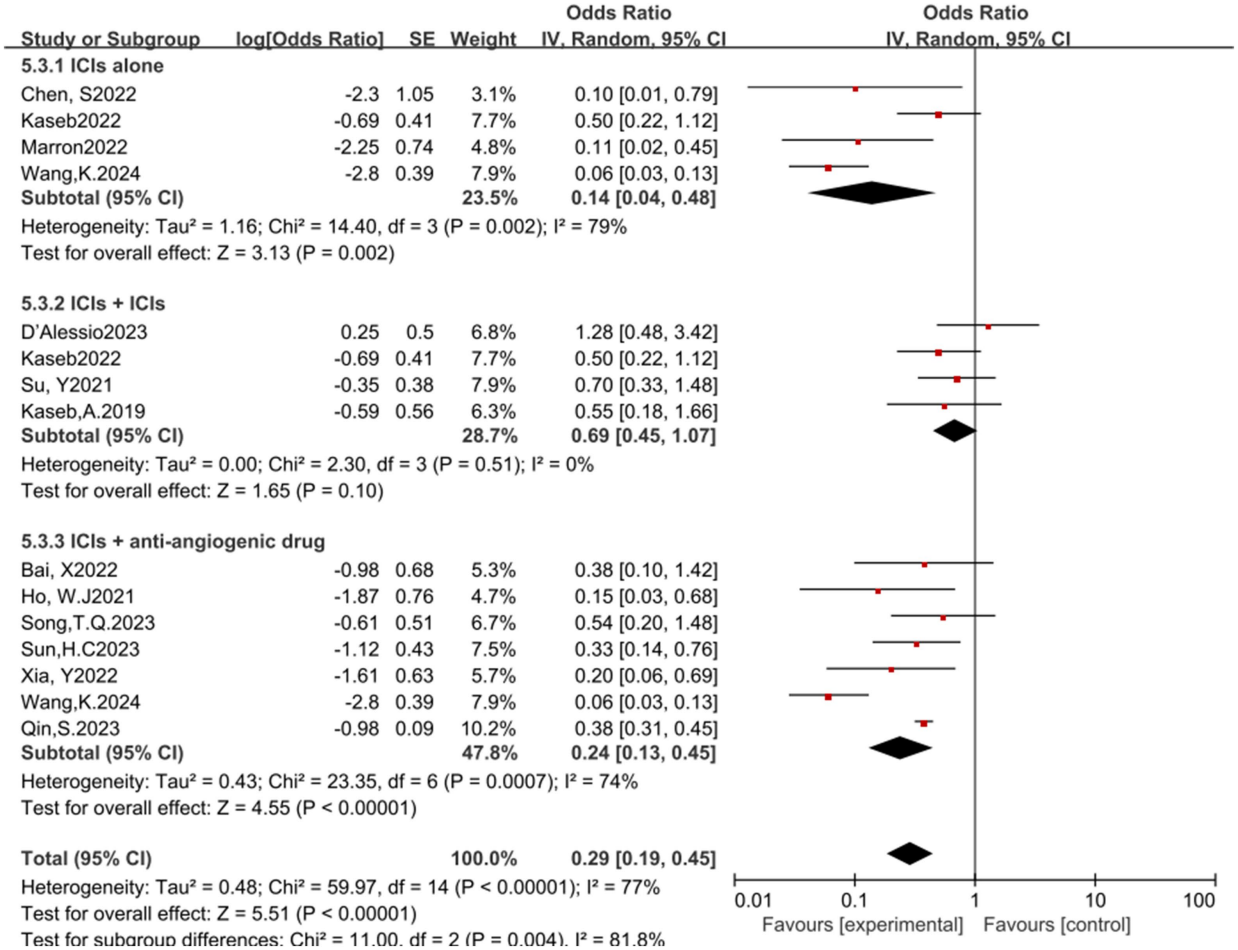
Subgroup analyses based on neoadjuvant immune checkpoint inhibitor combinations for Grade 3–4 TRAEs. TRAEs, treatment-related adverse events.

**Figure 12:**
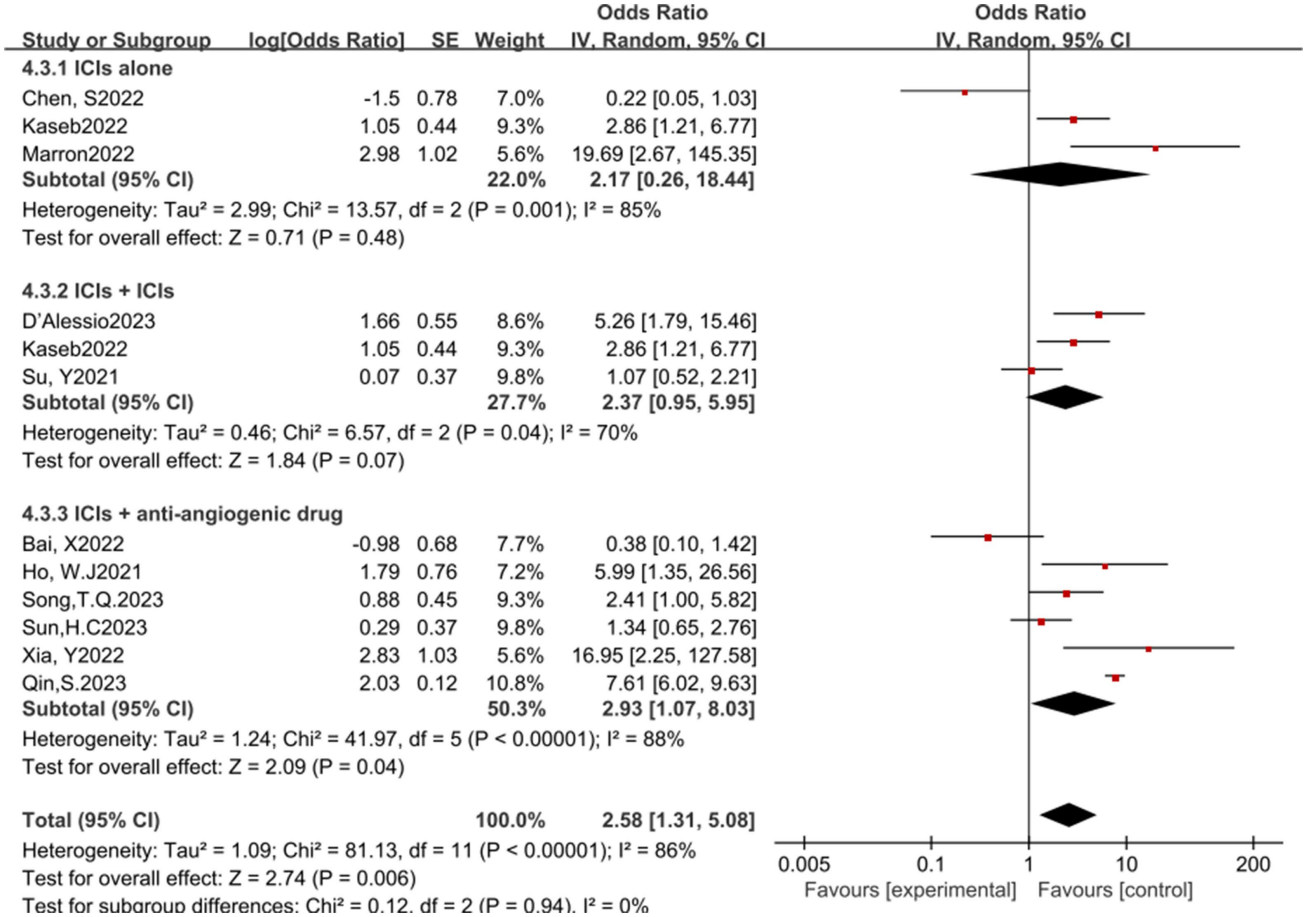
Subgroup analyses based on neoadjuvant immune checkpoint inhibitor combinations for Resection Rate.

In the analysis of various immunotherapeutic combinations, dual immune checkpoint inhibitor (ICI) therapy demonstrated a superior combined odds ratio (OR) for pathological complete response (pCR) compared to monotherapy (Figure 8), and monotherapy showed a higher combined OR than the combination of immunotherapy with targeted therapy, both exhibiting statistical significance. No significant differences were observed across groups for major pathological response (MPR) (Figure 9) and objective response rate (ORR) (Figure 10). Conversely, the combined OR for adverse events with dual ICI therapy was elevated compared to monotherapy, and monotherapy had a lower combined OR than the targeted therapy-immunotherapy combination, indicating significant inter-group variations (Figure 11). Surgical excision rates (Figure 12) remained consistent across all groups.

## 4 Discussion

The present study underscores the substantial therapeutic benefits of neoadjuvant immunotherapy in patients with resectable HCC, with a meta-analysis yielding an ORR of 0.37 (95% CI: 0.20–0.69). Notably, the highest ORR reported was 54.2% (18), underscoring the potential of neoadjuvant immunotherapy to enhance surgical outcomes. Corresponding pCR and MPR rates were 0.23 (95% CI: 0.14–0.37) and 0.47 (95% CI: 0.32–0.70), respectively, suggesting a promising role for this treatment modality in HCC management (24).

Despite the potential of neoadjuvant immunotherapy in improving surgical resection opportunities for liver cancer patients, current data on postoperative survival rates are limited. Most relevant studies are ongoing, leading to a scarcity of existing statistical data for assessing the long-term efficacy of neoadjuvant immune checkpoint inhibitor therapy in HCC. In this meta-analysis, only four studies provided specific statistical results. The study by Kaseb et al. (6) revealed that median progression-free survival (PFS) with nivolumab monotherapy was 9.4 months, with a 95% CI of 1.47 to not estimable (NE), whereas the PFS median significantly extended to 19.53 months with nivolumab combined with ipilimumab, with a 95% CI of 2.33 to NE. Additionally, other studies (15, 20) reported data on event-free survival (EFS) and recurrence-free survival (RFS). The median EFS was 13.8 months, with a 95% CI of 10.3 to 17.3, and the one-year RFS rate was 53.85%, with a 95% CI of 24.77 to 75.99%. However, due to the limitation of follow-up time, no studies have reported data on overall survival (OS) yet. These preliminary results suggest that neoadjuvant immunotherapy may have a positive impact on improving the prognosis of patients with HCC, but longer follow-up and more data are needed to fully assess its long-term effects on patient survival rates.

In studies of other tumor types, a significant correlation between pathological response and patient survival has been established (25). This study further explores this correlation in patients with HCC through similar statistical analysis. Ho et al. (14) found a correlation between achieving MPR and long-term disease-free survival (DFS). Currently, all patients have surpassed 230 days in DFS, suggesting the potential impact of pathological response on long-term patient prognosis. Kaseb et al. (6) also reported a positive effect of MPR on recurrence-free survival (RFS) and observed a significant difference (*p* = 0.049). Additionally, Xia et al. (15) noted higher RFS rates in patients achieving pCR or MPR, although this difference did not reach statistical significance, possibly due to the small sample size in the study. These results indicate that pathological response may be a potential predictor of prognosis in patients with HCC. However, to validate these preliminary findings, further research and a larger sample size are required to establish the exact relationship between pathological response and patient survival.

The safety assessment of neoadjuvant immunotherapy revealed an odds ratio (OR) of grade 3–4 treatment-related adverse events (TRAEs) of 0.27, with a 95% confidence interval (CI) ranging from 0.17 to 0.44. These immune therapy-related adverse reactions are mostly manageable, aligning with the findings from a recent study that evaluated the safety profile of immunotherapies in gastrointestinal tumors, which reported similar manageable adverse event profiles (26). In particular, a study on the combination therapy of nivolumab and ipilimumab (6) indicated a higher proportion of grade 3–4 TRAEs compared to monotherapy. However, the observed 20% difference (95% CI of 14.7 to 38.7%, *p* = 0.69) was not statistically significant, suggesting that different combination therapy regimens may elicit varied therapeutic responses and safety profiles. Therefore, optimizing drug use to maximize patient benefit should be a priority in future treatment strategies. Additionally, the overall surgical resection rate following neoadjuvant immunotherapy exhibited an OR of 3.91, with a 95% CI of 2.05 to 7.45. While the majority of patients proceeded with surgery as scheduled, a subset may lose surgical eligibility due to disease progression or face increased toxicity risks. Comprehensive preparation and management by medical teams for potential perioperative risks are crucial to maximize patient benefit and ensure treatment safety (20).

Subgroup analyses did not detect significant efficacy disparities among the three single-agent immune checkpoint inhibitors (ICIs). In contrast, dual ICI therapy showed superior efficacy compared to single-agent therapy, which in turn was more effective than the combination with targeted therapy. Interestingly, the rate of Grade 3–4 treatment-related adverse events (TRAEs) was notably lower with the combination of targeted therapy and immunotherapy, highlighting a reverse pattern in safety profiles. Despite the widespread clinical use of both targeted and immune therapies in HCC, monotherapy approaches may result in resistance and limited benefits, underscoring the potential advantages of combination treatments. The IMbrave150 trial (27) illustrated marked enhancements in both the one-year survival rate, which increased to 67.2%, and the median progression-free survival (mPFS), extended to 6.8 months, among untreated patients with advanced HCC who were treated with the combination of atezolizumab and bevacizumab. This regimen has been endorsed by the FDA and CSCO as a first-line therapy for advanced HCC. Additional regimens, including lenvatinib combined with pembrolizumab, sintilimab in conjunction with bevacizumab, and camrelizumab paired with apatinib, have demonstrated encouraging outcomes (28–31). At the mechanistic level, research indicates that immune-modulating agents help to reestablish a supportive immune microenvironment, whereas anti-VEGF therapies such as bevacizumab alleviate immunosuppression and promote vascular normalization, enhancing drug delivery. This strategy enables the use of lower doses of immune checkpoint inhibitors (ICIs), which in turn reduces the likelihood of adverse reactions (32, 33). In our subgroup analysis, the combination of targeted therapy and immunotherapy did not exhibit significant superiority. However, considering the limited follow-up duration, the potential for long-term survival benefits after surgery warrants further investigation, which could inform the selection of optimal treatment strategies in future clinical practice.

The application of immunotherapy in HCC treatment is burgeoning, with studies demonstrating significant long-term survival advantages for patients who respond to therapy (34). However, the challenge remains to identify patients who are more likely to benefit from treatment, a necessity underscored by the variable responses observed in clinical practice. In pivotal trials for primary liver cancer, such as CheckMate040 (35) and CheckMate459 (36), a PD-L1 expression threshold of 1% or higher was associated with improved median OS and ORR (35). Contrarily, the KEYNOTE224 (36) study found no significant correlation between tumor cell PD-L1 expression levels and treatment response rates (36). Beyond PD-L1, other potential biomarkers, including gut microbiota, circulating tumor DNA (ctDNA), and tumor-infiltrating lymphocytes, have been investigated for their predictive value in HCC treatment (37). However, these biomarkers require validation in large prospective studies to clarify their specificity and sensitivity. In this analysis, studies by Xia (15) and Ho (14) identified tumor-infiltrating B cells and dendritic cells (DCs) as potential biomarkers for antitumor immunotherapy. They noted that higher post-treatment DC levels correlated with a reduced risk of patient recurrence. D’Alessio (19) highlighted immune cell infiltration, peripheral cfDNA, and gut microbiota composition as predictive factors for the efficacy of the Nivolumab plus Ipilimumab regimen. Similar findings have been corroborated across various tumor types, including melanoma, colorectal cancer, and lung cancer (38). The ongoing development of precise and effective biomarker combinations or multidimensional predictive models aims to identify patient populations most likely to benefit from immunotherapy, thereby optimizing survival outcomes (8).

This study acknowledges its limitations, including inconsistencies across included studies, incomplete data sources, and a dearth of biomarker information, which restrict the analysis of long-term benefits of neoadjuvant therapy. Future research must address these limitations to more accurately assess the potential long-term impact of neoadjuvant therapy on patient prognosis.

## 5 Conclusion

Overall, neoadjuvant immunotherapy has shown effectiveness and safety in patients with resectable hepatocellular carcinoma. Despite observing positive pathological and radiological responses in some patients, which are associated with improved survival outcomes, the incidence of grade 3–4 adverse events is low. Future research needs to further validate these results to provide a stronger evidence base for the application of neoadjuvant immunotherapy in the treatment of hepatocellular carcinoma.
