# Correction: Meta-analysis of the efficiency and safety of neoadjuvant therapy with immune checkpoint inhibitors in resectable hepatocellular carcinoma

**DOI:** 10.3389/fmed.2025.1682417

**Published:** 2025-08-29

**Authors:** Frontiers Production Office

**Affiliations:** Frontiers Media SA, Lausanne, Switzerland

**Keywords:** neoadjuvant immunotherapy, hepatocellular carcinoma, meta-analysis, safety, efficiency

Authors Yadikaer Yasen and Xin Zhao were erroneously included as authors in the published article. The correct author list reads:

Adili Tuersun^1^, Munire Mohetaer^2^, Guanxin Hou^3^ and Gang Cheng^4^

^1^School of Life Sciences and Biopharmaceutics, Shenyang Pharmaceutical University, Shenyang, Liaoning, China

^2^School of Business Administration, Shenyang Pharmaceutical University, Shenyang, China

^3^Department of Pharmacy, General Hospital of Northern Theater Command, Shenyang, China

^4^Department of Pharmacy, Shenyang Pharmaceutical University, Shenyang, China

The Author Contributions Statement has been corrected to read:

